# UV Exposure Effects on Starch Films from an Ecuadorian Potato (*Solanum tuberosum*, *Chola* Variety): A Macro- and Nanoscale Investigation

**DOI:** 10.3390/polym18060720

**Published:** 2026-03-16

**Authors:** Cynthia Pico, Pablo Ilvis, Santiago Casado

**Affiliations:** 1 Facultad de Ciencia e Ingeniería en Alimentos y Biotecnología, Universidad Técnica de Ambato, Ambato 180207, Ecuador; cl.pico@uta.edu.ec (C.P.); pilvis9638@uta.edu.ec (P.I.); 2 Group for Universal Advance in bioScience (GUAIS), Universidad Técnica de Ambato, Ambato 180207, Ecuador

**Keywords:** starch films, potato starch, atomic force microscopy, UV irradiation, biopolymers

## Abstract

The growing pollution caused by plastics with slow degradation kinetics is demanding the search for biodegradable alternatives. Starch-based films are a promising option, but their practical application may be limited by their potential susceptibility to rapid ultraviolet (UV) exposure degradation. This study evaluates the effect of prolonged UV-C irradiation (254 nm, 168 h) on plasticizer-free films derived from the starch of an Ecuadorian potato *Solanum tuberosum* (*Chola* variety). Films formulated at 3% and 5% (*w*/*v*) starch were characterized before and after UV exposure. The analysis includes the evaluation of optical, mechanical, and physicochemical properties, along with Fourier Transform Infrared spectroscopy (FTIR) and atomic force microscopy (AFM) for nanoscale surface inspection. UV irradiation increased the opacity of the films but reduced slightly their tensile strength, elongation at break, moisture content, and total soluble matter. In contrast, the elastic modulus remained relatively high. FTIR analysis revealed no significant formation of new functional groups. AFM measurements indicated that irradiation caused only minor nanoscale alterations in the same film regions. These alterations were more pronounced in films with higher starch concentrations. The results demonstrate that UV-C exposure induces minor structural adjustments in plasticizer-free starch films derived from the *Chola* variety, without compromising their fundamental integrity. Consequently, this work advances the understanding of the environmental stability of these films and supports their potential application as sustainable materials, even in conditions involving UV exposure.

## 1. Introduction

Environmental plastic pollution currently represents one of the most complex and persistent challenges [1,2,3,4]. Organic waste polymers can degrade in a few days at ambient conditions, whereas a common piece of petroleum-derived plastic may persist in the environment for up to five hundred years [5]. Several studies have shown that during degradation, these substances release micro-fragments that accumulate in numerous ecosystems and in animals across the planet [6,7,8]. It is estimated that approximately 25.3 million metric tons of plastic waste enter the oceans each year, of which about 16.8 million metric tons sink to the seabed, 6.6 million metric tons float as macroplastics, and 1.8 million metric tons accumulate on coastlines [9,10]. Micro- and nanoplastics have been identified not only as ubiquitous emerging pollutants but also as potential vectors for toxic substances [11]. Their capability of crossing biological barriers and altering physiological processes has generated increasing scientific concern regarding their ecotoxicological implications [12,13,14].

These findings have intensified the search for sustainable, biodegradable alternatives that can reduce environmental impact while maintaining adequate performance. Biopolymers derived from renewable and abundant resources have been proposed as alternatives. Among these, starch has emerged as a promising substitute for conventional petroleum-based plastics due to its biodegradability and wide availability [15,16]. Their potential applications span the packaging, biomedicine, and agriculture sectors due to their transparency, barrier properties, and compatibility with biodegradable processing methods [17,18]. However, their behavior under ambient conditions and the limited mechanical strength of some biodegradable films may restrict their wider industrial application [18,19].

The mechanical and physicochemical properties of starch-based films are strongly influenced by their molecular structure, the preparation method, and the botanical source, with significant variations even among different varieties of the same species [20,21,22,23]. Ecuadorian potato (*Solanum tuberosum*) starch extracted from the *Chola* variety has been shown to produce films with higher strength and rigidity than those generated from other starch sources [20,24,25]. However, the environmental stability of these films when exposed to abiotic stress factors, such as ultraviolet (UV) irradiation, remains largely unexplored, particularly in systems without plasticizers. Previous studies have reported that UV light can alter hydrogen bond networks, modify surface morphology, and affect the mechanical performance of polymeric films, depending on the material composition, UV exposure conditions, and wavelength [26,27]. More specifically, it has been proven that UV-C radiation causes photo-oxidation and polymer chain scission in several bio-based polymeric systems [28,29].

This study evaluates the effect of prolonged exposure to UV-C light (254 nm) on films prepared from the starch extracted from potato (*S. tuberosum*, *Chola* variety) at two different concentrations and without plasticizer. The films were characterized before and after UV irradiation using a combination of macro-, nano-, and chemical analysis techniques, including opacity assays, mechanical testing, Fourier-Transform Infrared (FTIR) spectroscopy, as well as solubility, moisture content, and Atomic Force Microscopy (AFM) measurements. Although clear differences were observed, the results demonstrate that irradiation leaves the core film structure largely intact, preserving its fundamental properties despite a slight reduction in efficacy.

## 2. Materials and Methods

### 2.1. Starch Extraction

Potato starch was extracted from an Ecuadorian potato (*S. tuberosum*), *Chola* variety. The extraction procedure followed the method proposed by Pico et al. [24]. The potatoes were washed and cut into small pieces and subsequently ground. Distilled water (1 L) was added to the resulting slurry, which was then filtered through gauze with an approximate pore size of 120 mesh. The filtrate was allowed to stand for 6 h to facilitate starch sedimentation. Subsequently, the supernatant was discarded, and the precipitate was dried in an oven at 45 ± 5 °C for approximately 12 h. The starch yield, expressed as a weight percentage relative to the fresh potato mass, was 13%.

### 2.2. Film Preparation

Potato starch films were prepared by dispersing 3 and 5 g of starch in 100 mL of distilled water. Initially following the methodology described by Farhan and Hani [30], the suspensions were heated to 90 °C using a hot plate with magnetic stirring at a constant speed of 200 rpm. Subsequently, the samples were centrifuged at 7000 rpm and 25 °C for 15 min. The resulting supernatant was poured into 100 × 10 mm Petri dishes at a volume of 20 mL per dish. The plates were then dried in an incubator at 45 °C for 24 h and finally stored in a desiccator at room temperature.

### 2.3. Optical Characterization

The visual characteristics and apparent transparency of the films were assessed by placing specimens over a patterned background containing black text and figures. Digital images were captured under diffused white light using a standard digital camera.

For microscopic analysis, square segments of approximately 10 mm per side from each starch-based film were excised and examined using an EVOS XL microscope (Life Technologies, Thermo Fisher Scientific, Waltham, MA, USA). Samples were attached to glass slides using adhesive tape, oriented with the film surface facing downward, and visualized under transmitted white light. Observations were performed using a 40× air-immersion objective with a numerical aperture of 0.65.

### 2.4. Mechanical Properties Measurement

Elastic properties of the films, including tensile strength, elastic modulus, and elongation at break, were evaluated at ambient conditions following a procedure adapted from the ASTM D 882-88 standard method [31]. A Brookfield CT3 texture analyzer (Ametek, Berwyn, PA, USA) was employed for these mechanical tests. Specimens were prepared by cutting the films into rectangular strips measuring 50 mm in length and 20 mm in width. The crosshead speed was set at 0.5 mm/s. For each sample, the reported values represent the mean calculated from 7 replicate measurements.

### 2.5. Fourier Transform Infrared Spectroscopy (FTIR)

Fourier transform infrared (FTIR) spectroscopy was employed following the method proposed by Orsuwan et al. [32]. A Spectrum Two spectrometer (Perkin-Elmer, Waltham, MA, USA) equipped with an attenuated total reflectance (ATR) accessory was used. Spectra were recorded over a range of 500 to 4000 cm^−1^ at a resolution of 4 cm^−1^. Film specimens were placed directly on the ATR crystal surface at ambient laboratory conditions, and were gently pressed with the flat-tip plunger. For each sample, spectra were collected in triplicate from different locations to ensure representativeness.

### 2.6. UV–Visible Optical Absorption and Opacity

The optical opacity of the films was determined using an accuSkan GO UV-Vis spectrophotometer (Thermo Fisher Scientific, Waltham, MA, USA). Rectangular specimens, sized to fit within the instrument’s quartz cuvettes, were cut from the film samples for spectral analysis. Measurements were performed in triplicate under ambient laboratory conditions across the 200–600 nm wavelength range. Opacity (*O*) was calculated using Equation (1), where *Abs*_560_ represents the absorption at a wavelength of 560 nm.

(1) $$O=\frac{{Abs}_{560}}{X}$$

A micrometer caliper, with 0.01 mm accuracy, was employed to measure the film thickness (*X*, mm). Data were averaged from eight different locations on each film.

### 2.7. Moisture Content (MC) Determination

For the determination of the moisture content (MC), a gravimetric method was employed using an analytical balance with a precision of 0.01 mg. Specimens of comparable dimensions were weighed without any prior preparation, and values were recorded as the initial mass (*W*_0_). The samples were subsequently subjected to drying in an oven maintained at 105 °C for 24 h. Following the drying period, they were transferred to a desiccator containing silica gel and allowed to cool to ambient temperature. Once acclimatized, they were reweighed, and values were recorded as the final mass (*W_f_*). Each measurement was conducted in triplicate. The moisture content (*MC*) was then calculated using Equation (2).

(2) $$MC=\frac{W_{0}-W_{f}}{W_{0}}\times 100$$

### 2.8. Total Soluble Matter (TSM) in Water

Total Soluble Matter (TSM), also referred to as water solubility, was determined following the method described by Arancibia et al. [33] and Salazar et al. [34]. Film specimens were cut into square pieces of approximately 5 mm per side. Their initial mass (*W_i_*) was recorded using an analytical balance with a precision of 0.01 mg. Each sample was then immersed in an individual flask containing 30 mL of distilled water. The flasks were placed on an orbital shaker set at 70 rpm and maintained at room temperature for 24 h. After this period, the samples were recovered and dried in an oven at 105 °C for 24 h. Subsequently, the final mass (*W_f_*) of each sample was measured, and their TSM content was calculated using Equation (3).

(3) $$TSM=\frac{W_{i}-W_{f}}{W_{i}}\times 100$$

### 2.9. Atomic Force Microscopy (AFM) Characterization

Surface characterization of each starch film at the nanoscale was performed using the Park System XE7 Atomic Force Microscope (Santa Clara, CA, USA), following the methodology described by Ilvis et al. [20]. A sample from each starch film was cut and mounted onto the AFM magnetic sample holder using double-sided adhesive tape. The measurements were performed in tapping mode under ambient conditions using NCHR cantilevers (nominal spring constant of 42 N/m, resonance frequency of 320 kHz, and tip diameter < 10 nm). This dynamic mode (tapping) was selected because it has proven effective for scanning microscopic structures covered with loose and sticky debris [35], a condition potentially present on the starch-based films after irradiation. The scan resolution was maintained at 512 × 512 pixels. Atomic Force Microscopy (AFM) images were processed using XEI software version 5.1.6 (Park Systems, Santa Clara, CA, USA), applying linear background subtraction.

### 2.10. UV Exposure

The processed films were conditioned at a temperature of 23 ± 2 °C and at a relative humidity of 50 ± 5% for 48 h. The films were then placed at a distance of 20 ± 2 cm from the irradiation source and irradiated using a UVP Sterilaire XX-20S lamp (UV-C, 254 nm, 20 W) for 168 h of continuous exposure. The irradiance in the plane of the samples was approximately 0.2 mW/cm^2^, corresponding to a total radiation dose around 121 J/cm^2^ for the duration of the experiment (i.e., 168 h or 7 days). Other studies have shown that smaller UV-C doses are able to induce measurable structural and physicochemical changes in certain bio-based polymers, including molecular chain rearrangements [28,29]. Control samples were maintained under identical environmental and temporal conditions without UV exposure. This was achieved by placing the control samples close to the lamp where the irradiated samples were exposed, taking special care to block the UV light completely from reaching the control samples. After irradiation, the samples were reconditioned prior to mechanical, physicochemical, and surface characterization.

### 2.11. Statistical Analysis

Statistical analysis of the data was performed using two-way ANOVA, followed by Tukey’s *post hoc* test to identify significant differences between means. All tests were performed at a 95% confidence level (*p* < 0.05). Analyses were conducted using the software R version 4.4.0 (R Development Core Team, Vienna, Austria).

## 3. Results and Discussion

### 3.1. Optical Characterization of the Films

**Figure 1:**
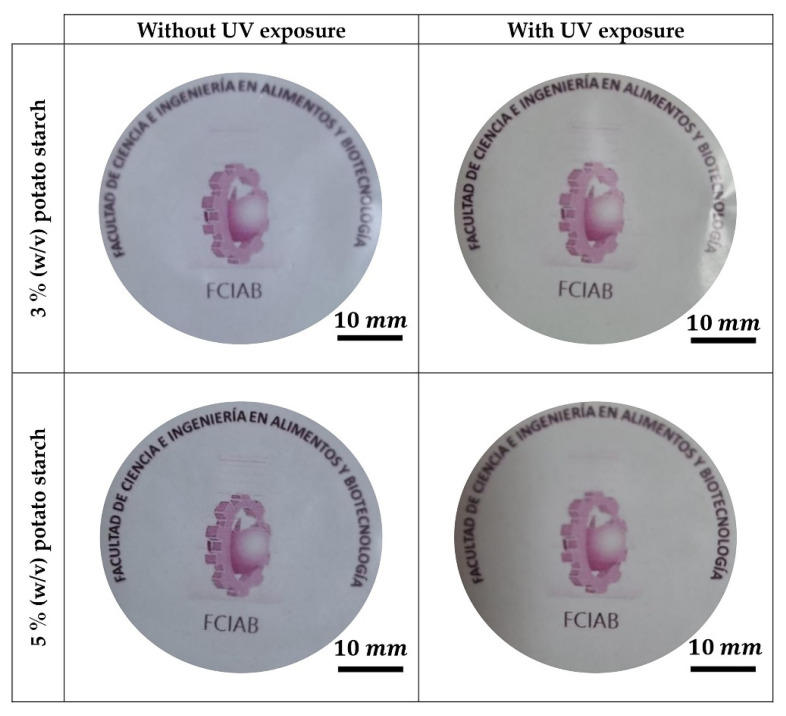
Apparent transparency of *Chola* potato starch films with and without UV exposure. Text and image transmitted through the films correspond to our University department.

The apparent transparency of the films prepared from starch extracted from the *Chola* potato variety is shown in Figure 1. Control starch films showed significantly higher transparency than the UV-irradiated films. The higher transparency of the control films is typical of potato starch-based materials, possibly due to factors that favor greater light transmittance and optical clarity, such as the morphology of the starch granules, high swelling capacity, and phosphate monoester content [36]. In contrast, when the films were exposed to UV irradiation, they became visibly more opaque, with a yellowish color.

This increase in the apparent opacity may be related to structural changes occurring on the surface of the films, associated with chemical processes such as a different entanglement of molecules during exposure. UV light could induce bond breakage and generate free radicals in the outer layer of the films, which may subsequently recombine to form covalent bonds, reducing molecular mobility and resulting in a more rigid and compact polymeric structure, as has been described for some polymeric films exposed to UV radiation [37,38,39].

**Figure 2:**
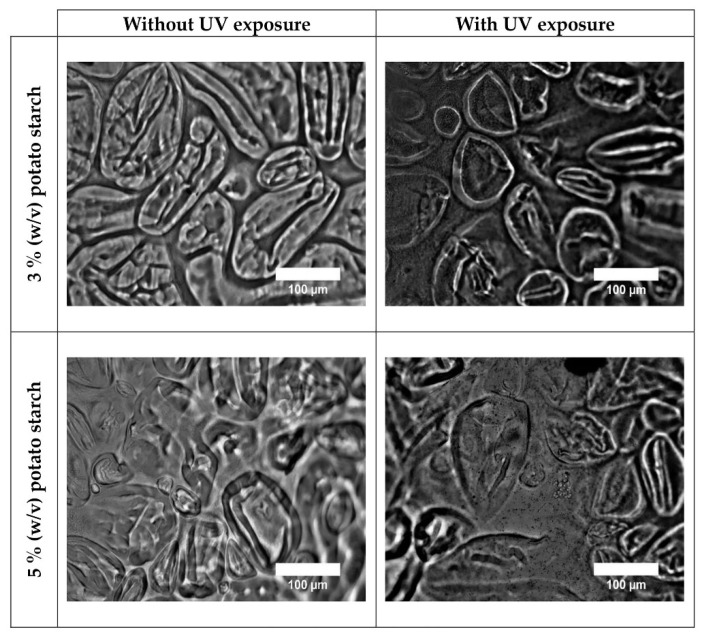
White light transmission optical microscopic images of *Chola* potato starch film samples with and without UV exposure.

Optical microscopy using white transmitting light revealed differences between the structures of each sample, as shown in Figure 2. Films not irradiated with UV light showed a more transparent aspect, with lower apparent opacity and higher intensity contrast, compared to the films exposed to UV radiation. The observed increase in the homogeneity between the interior and exterior of the grains could potentially be attributed to sample dehydration after UV irradiation. These observations are consistent with previous reports on starch-based films, where increased matrix density and structural rearrangements decrease light transmission following external treatments [40].

### 3.2. Mechanical Properties

**Figure 3:**
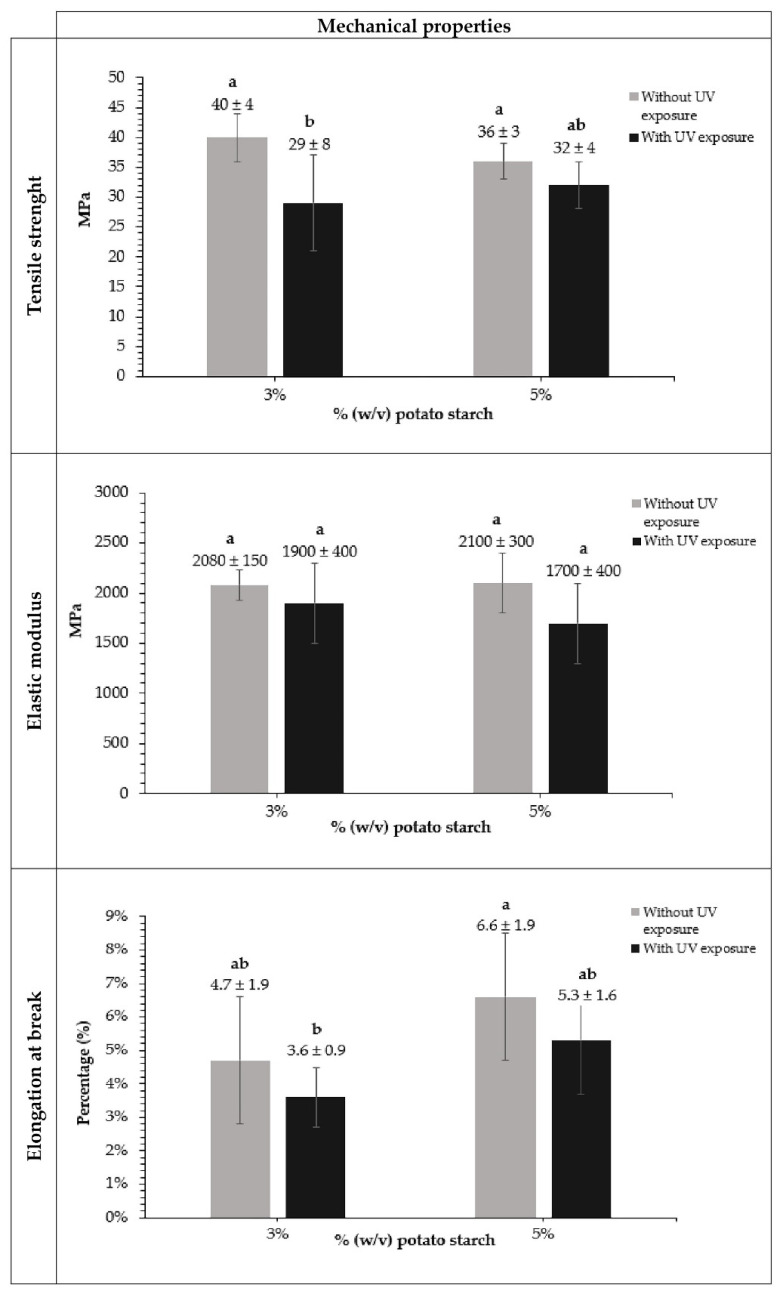
Mechanical properties of potato starch films with and without UV exposure, at different starch concentrations. The data shown are representative of seven independent experiments and comprise each mean ± SD. Different letters (a, b) indicate significant differences between the different films (*p* ≤ 0.05).

Figure 3 compares the tensile strength, modulus of elasticity, and elongation at break of the films with varying *Chola* potato starch concentration, both before and after exposure to UV radiation.

Films not exposed to UV radiation exhibited higher tensile strength (40 ± 4 MPa and 36 ± 3 MPa for 3% and 5% (*w*/*v*) potato starch, respectively). After UV exposure, these values decreased to 29 ± 8 MPa and 32 ± 4 MPa for 3% and 5% (*w*/*v*) potato starch, respectively. The values without irradiation were similar to those reported by Ilvis et al. [20] and higher than those reported by Dutta and Sig [41]. Films with higher starch concentrations showed a smaller difference, which could be related to the fact that starch forms a polymer network with greater density and stability [42]. Ilvis et al. [20] mention that the *Chola* variety potato has a high amylose content, which could contribute to greater strength in the polymer network. Furthermore, during the film manufacturing process, the starch solution was centrifuged after gelatinization to enrich the supernatant with a higher amount of amylose.

The elastic modulus obtained before irradiating the films was 2080 ± 150 MPa and 2100 ± 300 MPa for the films with 3% and 5% (*w*/*v*) potato starch, respectively, indicating that the films exhibit high initial stiffness. These values are similar to those reported by Ilvis et al. [20] for *Chola* potato starch and higher than those reported by Pico et al. [24] and Domene Lopez et al. [43] for starch extracted from different potato varieties. The 3% (*w*/*v*) starch films exposed to UV irradiation showed slight changes in the elastic modulus (from 2080 ± 150 MPa to 1900 ± 400 MPa), while the 5% (*w*/*v*) starch films showed a moderate decrease from 2100 ± 300 MPa to 1700 ± 400 MPa. At both concentrations, a high value was maintained after UV light exposure, suggesting that the polymer network retains its overall structural stability.

The elongation at break for 3% and 5% (*w*/*v*) starch content films before exposure was 4.7 ± 1.9% and 6.6 ± 1.9%, respectively. These values are similar to those revealed by Ilvis et al. [20] for the potato variety *Chola* without glycerol, where elongations close to 6% were reported, attributed to the amount of amylose and a compact polymeric network. In contrast, studies by Dai, Zhang, and Cheng [44] show elongations in a range of 46% to 51.66%, although in this case glycerol was added. In this particular research, more ductile and less rigid films were obtained, possibly due to the addition of the plasticizing agent, which reduces the rigid intermolecular interactions and increases the mobility in the polymeric chains. After exposure to UV irradiation, the elongation at break shows a minor descent, resulting in a slightly more brittle film. These small structural changes could be caused by chain scissions, the formation of free radicals or crosslinking, but also could be attributed to the dehydration of the samples during the exposure. In any case, the main network structure is apparently preserved.

### 3.3. Fourier Transform Infrared Spectroscopy (FTIR) Absorption

**Figure 4:**
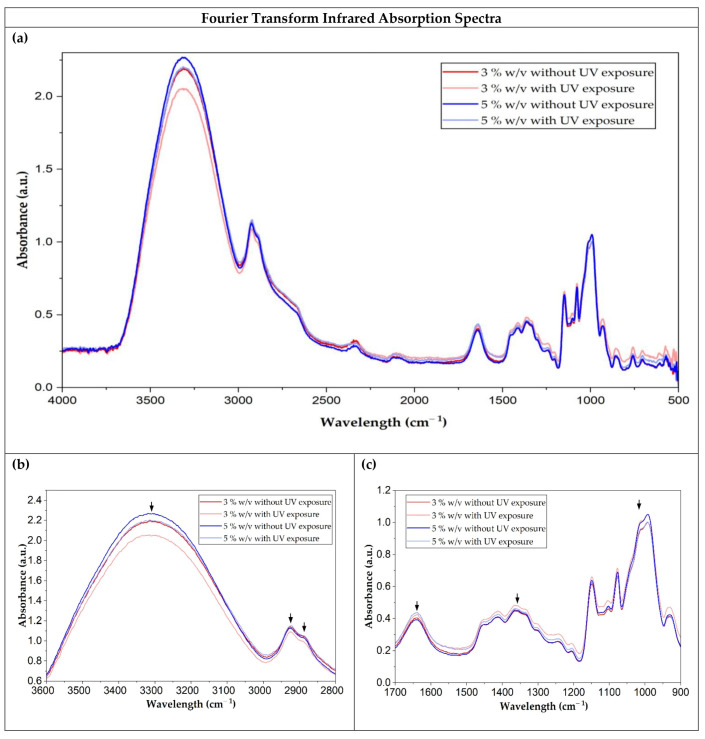
Fourier transform infrared absorption spectra of the films in the 4000–500 cm^−1^ range (**a**). Expanded views of the 3600–2800 cm^−1^ (**b**) and 1700–900 cm^−1^ (**c**) regions. Arrows highlight representative bands.

Fourier transform infrared spectroscopy (FTIR) permits the observation of characteristic bands corresponding to specific chemical bonds present in each film. The spectrograms obtained with and without UV exposure are shown in Figure 4.

The spectra obtained at different starch concentrations without UV exposure are similar to the characteristic bands obtained previously [20,24]. The region between 3600 cm^−1^ and 3200 cm^−1^ is attributed to the stretching of the hydroxyl (OH) groups present in the starch chains and absorbed water [45]. This region shows sensitivity to moisture content, indicating the existence of a hydrogen bond network that maintains the film structure [20]. A slight reduction is observed in this band at both concentrations, which could be a sign of possible dehydration during the exposure.

The region between 2940 cm^−1^ and 2840 cm^−1^ is attributed to C-H stretching vibrations, corresponding to the main chains of the starch polymer [46]. Furthermore, the band around 1638 cm^−1^ represents bending vibrations of the absorbed water, while the bands between 1390 cm^−1^ and 1380 cm^−1^ are related to bending vibrations of the COH group [47].

The bands between 1200 cm^−1^ and 900 cm^−1^ correspond to the stretching vibrations of C-O, C-C and COH, in the starch structure [48]. A slight change in these bands was observed; more noticeable in the films made with 5% (*w*/*v*) starch. This could be related to a higher density of intermolecular bonds in the matrix, which facilitates the reorganization of the structures upon interaction with UV radiation [49].

Bajer, Kaczmarek, and Bajer [29] and Gutiérrez-Silva [50] reported that UV irradiation of films from different sources and in the absence of plasticizers leads to modifications in the intensity of the hydroxyl bands in the 3200–3400 cm^−1^ region and alterations in the fingerprint region. Similarly, Shahabi, Goudarzi, and Babaei [51] reported that starch films containing plasticizers exhibit spectral changes associated with hydrogen bond reorganization and water loss, without evidence of intense oxidative phenomena. These results suggest that UV irradiation mainly induces a physical reorganization of the polymer network in starch films prepared at different concentrations and in the absence of plasticizers, with very limited generation of new functional groups.

### 3.4. Optical Absorption

**Figure 5:**
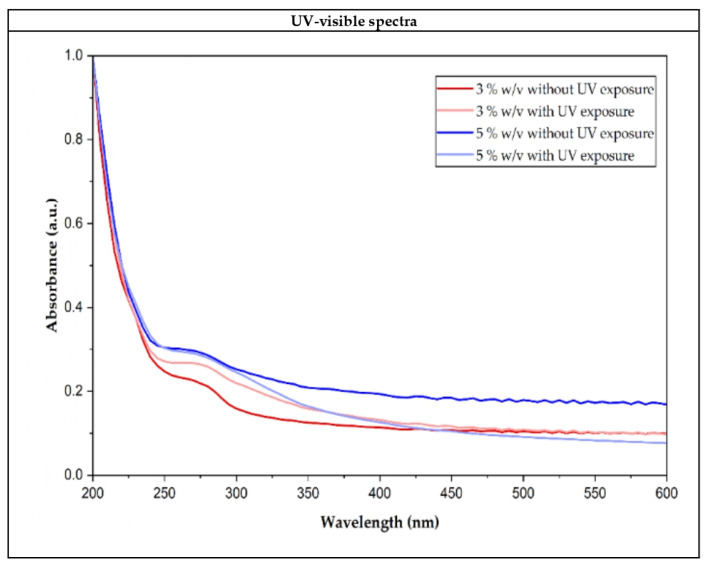
UV-Vis spectra of starch films prepared from 3% and 5% (*w*/*v*) solutions, before (without) and after (with) UV exposure.

Figure 5 shows the spectra obtained at 200–600 nm from starch films at different starch percentages with and without UV exposure.

The UV-Vis spectrophotometric analysis of potato starch films reveals high absorbance values in the UV region (200–250 nm) and a progressive decrease towards the visible region (300–600 nm). When comparing samples exposed and not exposed to UV irradiation, an increase in absorbance was observed in the irradiated films, particularly in the UV and near-UV regions. This suggests the possible formation of species with greater absorption or reflection capacity in this region of the spectrum, which could be related to photo-induced processes in the polymer matrix, such as oxidation phenomena or a rearrangement of the existing functional groups.

Thickness of all the films explored in this analysis is shown in Table 1.

Table 2 presents the opacity values obtained through the thickness indicated in Table 1 and the absorbances measured at 560 nm.

At both starch concentrations of 3% and 5% (*w*/*v*) the opacity values before UV irradiation are low and show no significant differences between them (1.00 mm^−1^ and 0.83 mm^−1^, respectively), indicating high transparency at this wavelength. This behavior could be related to a relatively homogeneous polymer structure that allows for efficient light transmission through the films. Previous studies on starch-based edible films have demonstrated that starch concentration plays a key role in determining optical properties by influencing matrix homogeneity and light scattering behavior [20,52]. After the films are exposed to UV light, a significant increase in opacity is observed in the two starch concentrations analyzed.

This effect may be attributed to UV-induced cleavage of glycosidic bonds and partial oxidation of starch chains, leading to the formation of free radicals, which act as centers for light absorption or scattering, thereby reducing optical transmittance [39]. In addition, UV irradiation may promote molecular rearrangements and local variations in film density, generating internal heterogeneities that enhance light reflection and scattering within the films.

### 3.5. Moisture Content (MC)

Moisture content results are shown in Table 3.

The moisture content at concentrations of 3% and 5% (*w*/*v*) without exposure to UV irradiation showed low values (11.5% and 11.6%, respectively). This indicates that the films exhibit high cohesion of the polymer matrix and a lower water retention capacity, possible due to structural compaction and reduced mobility of the starch chains. The values obtained are similar to those reported previously [20,53].

When the films were exposed to UV light, the moisture content decreased significantly to 7.1% for films with 3% (*w*/*v*) starch concentration and to 6.8% for films with 5% (*w*/*v*). This could be attributed to photo-dehydration and possible hydrogen bond network reorganization processes that reduce the films’ water absorption capacity. Previous studies have reported that in thermoplastic starch films, UV irradiation causes oxidation and chain cleavage, reducing the hydroxyl sites available to form hydrogen bonds with water [37,54]. These experimental values are in agreement with the decrease in the absorption band around 3300 cm^−1^ observed in the FTIR measurement (Figure 4b). The study published by Uyarcan and Güngör [55] also reports that UV light decreases surface moisture and hygroscopic capacity of starch-based films, by losing bound water and undergoing slight structural shrinkage, which results in drier films that are more stable against ambient moisture absorption.

### 3.6. Water Total Soluble Matter (TSM)

Table 4 shows the total soluble matter content in water measured on the samples.

TSM of films without UV exposure did not vary significantly with starch percentage and exhibited high values, similar to those obtained previously [20]. A high TSM value indicates low crystallinity, probably influenced by the amylose/amylopectin ratio of the starch. After UV exposure, TSM decreases at both starch concentrations, indicating that the films exhibit greater internal cohesion and lower solubility. According to Quispe, López, and Villar [37] and Uyarcan and Güngör [55], UV exposure induces a mild oxidation and a crosslinking process, resulting in a denser and less soluble structure. The observed decrease in TSM suggests the formation of a polymer network with greater stability against hydrolytic degradation processes, confirming the MC analysis.

### 3.7. Nanoscopic Characterization

**Figure 6:**
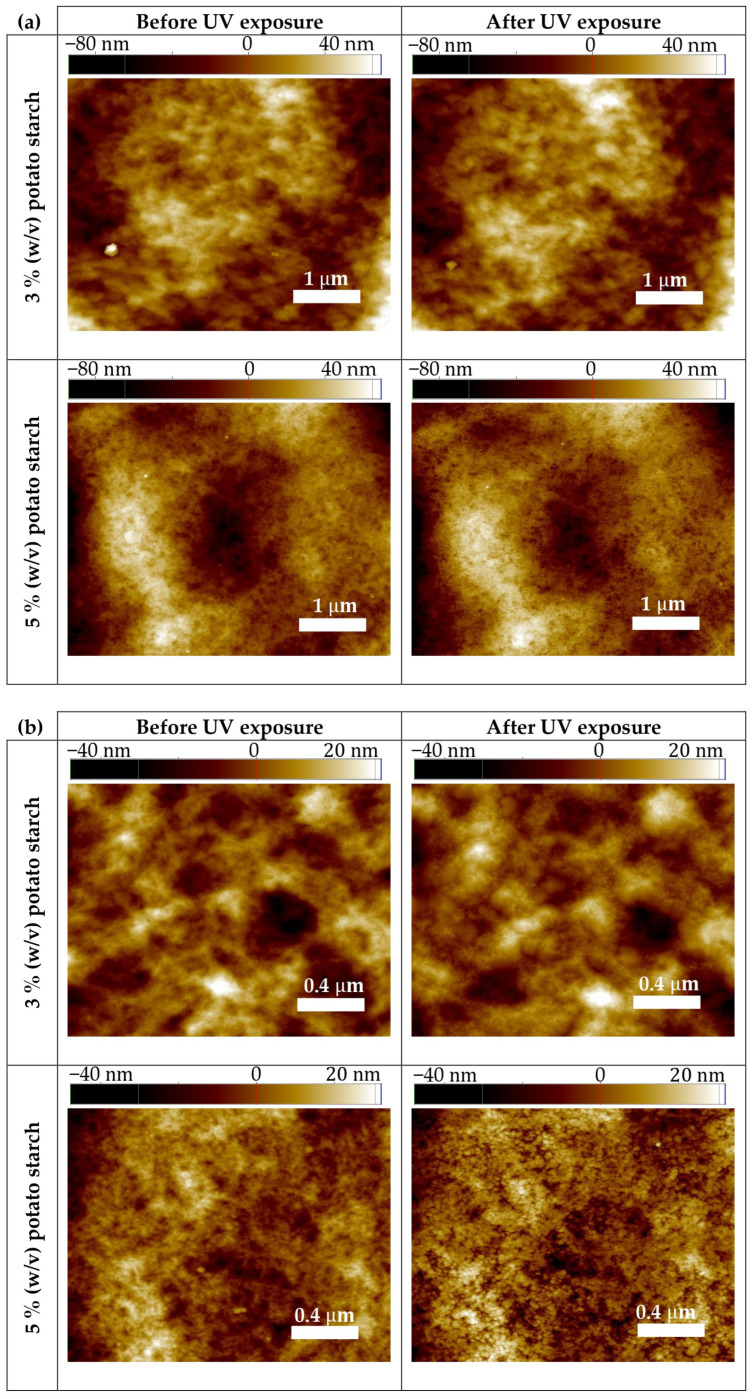
Atomic Force Microscopy (AFM) topographic images in tapping mode obtained on the surface of films from each potato starch concentration, before and after exposure to UV irradiation in the same regions at scan sizes of 5 × 5 µm^2^ (**a**) and 2 × 2 µm^2^ (**b**).

Figure 6 shows Atomic Force Microscopy (AFM) images acquired in tapping mode, presenting a comparison of the surface topographies in the same areas of the potato starch films with starch concentrations of 3% and 5% (*w*/*v*), both with (after) and without (before) UV exposure, at two different scan sizes. All films without UV exposure showed a predominantly granular morphology, probably associated with the presence of domains derived from partially gelatinized starch granules used in their preparation, which generates a micro-rough surface characteristic of polysaccharide-based matrices after drying [43,56]. Surface topography became more heterogeneous in the 5% (*w*/*v*) samples, with more pronounced elevations and depressions, probably caused by a higher density of amylose and amylopectin chains, which promotes intermolecular aggregation during the drying process. Nevertheless, despite this granular structure, surfaces appear quite united at this scale in all the cases analyzed.

After UV exposure, exactly the same regions were found and measured again, which allows the comparison of the topography of the surface at the nanoscale. This enables the detection of modifications caused solely by the UV-C light, rather than by the intrinsic roughness heterogeneity of the samples. Slight changes in the surface topography are observed at both concentrations and span ranges, although the main structure was maintained in all cases. Clearer differences are detected on the films prepared with 5% (*w*/*v*) starch concentration at the smallest scan size analyzed, showing more separated protrusions. This suggests that higher starch concentrations may promote UV energy absorption and amplify structural changes within the polymer matrix, perhaps due to a local structural reorganization and partial crosslinking processes when subjected to UV radiation.

To further corroborate the minor differences found between AFM topographic images before and after irradiation, a roughness comparison is performed. Choosing the Root Mean Square (RMS, Rq) parameter, a small increase in roughness is observed in all cases analyzed (Table 5), confirming the previous observation. This suggests that the topographic modifications induced by UV irradiation only promote low magnitude topographic adjustments, without causing significant structural alterations on the surface.

## 4. Conclusions

Starch-based films without plasticizers, derived from an Ecuadorian potato (*S. tuberosum*, *Chola* variety) at two different concentrations, were analyzed both before and after prolonged UV-C irradiation (wavelength: 254 nm). The study systematically evaluated their physicochemical, mechanical, and nano-structural properties. The findings revealed that UV exposure induces several minor modifications in these films, although the core structure preserves its fundamental integrity.

At the macroscopic level, irradiation led to a marked increase in film opacity and the development of a yellowish coloration, indicating reduced light transmittance that may be associated with structural rearrangements within the polymeric matrix. However, although the treated films exhibited a minor decrease in tensile strength and elongation at break, their elastic modulus remained relatively high, suggesting that the starch network preserves its overall structure, despite becoming slightly more brittle.

FTIR spectroscopy confirmed that UV light does not generate significant new functional groups. The observed reduction in moisture content and total soluble matter indicates a decrease in hydrophilic interactions, which could be attributed to factors such as an internal densification of the polymer network. This suggests an enhanced resistance to water uptake and dissolution after UV irradiation.

At the nanoscale, the same surface topography was inspected on each sample before and after UV exposure using AFM. Moderate changes were detected, which were clearer for the most concentrated sample. However, although an increase in surface heterogeneity and roughness was observed, the primary structure remained largely intact even at this scale.

In conclusion, these promising plasticizer-free starch-based films, derived from this Ecuadorian potato (*S. tuberosum*, *Chola* variety), have proved exceptional performance not only at ambient conditions but also under UV-C irradiation. These findings provide valuable insights into the search for ecological films and highlight their potential for use in sustainable applications where exposure to ultraviolet radiation is unavoidable.

## Figures and Tables

**Table 1:** Thickness (µm) of each film with different percentages of potato starch with and without exposure to UV irradiation.

% (*w*/*v*) Potato Starch	Treatment	Thickness (µm)
3	Without UV exposure	82 ± 23 ^ab^
3	With UV exposure	71 ± 15 ^b^
5	Without UV exposure	114 ± 14 ^a^
5	With UV exposure	113 ± 12 ^a^

Different letters (a, b) within the column indicate significant differences (*p* ≤ 0.05) according to Tukey’s test. Data shown correspond to the measurements performed with eight replicates and represent the mean ± SD.

**Table 2:** The opacity measured on potato starch films with and without UV irradiation.

% (*w*/*v*) Potato Starch	Treatment	Opacity (AU/mm)
3	Without UV exposure	1.00 ± 0.02 ^b^
3	With UV exposure	2.12 ± 0.02 ^a^
5	Without UV exposure	0.83 ± 0.02 ^b^
5	With UV exposure	2.41 ± 0.02 ^a^

Different letters (a, b) within the column indicate significant differences (*p* ≤ 0.05) according to Tukey’s test. Data shown correspond to the measurements performed with three replicates and represent the mean ± SD.

**Table 3:** Moisture content (MC) of potato starch films with and without UV irradiation.

% (*w*/*v*) Potato Starch	Treatment	Moisture Content (%)
3	Without UV exposure	11.5 ± 0.5 ^a^
3	With UV exposure	7.1 ± 0.8 ^b^
5	Without UV exposure	11.6 ± 0.6 ^a^
5	With UV exposure	6.8 ± 0.6 ^b^

Different letters (a, b) in the same column indicate significant differences between the different films (*p* ≤ 0.05). Data shown correspond to the measurements performed with three replicates and represent the mean ± SD.

**Table 4:** Total Soluble Matter (TSM) in water for films prepared with two starch concentrations, measured with and without UV irradiation of the films.

% (*w*/*v*) Potato Starch	Treatment	TSM (%)
3	Without UV exposure	28.1 ± 0.6 ^a^
3	With UV exposure	17.7 ± 0.8 ^b^
5	Without UV exposure	29.2 ± 0.6 ^a^
5	With UV exposure	18.6 ± 1.0 ^b^

Different letters (a, b) in the same column indicate significant differences between the different films (*p* ≤ 0.05). Data shown correspond to the measurements performed with three replicates and represent the mean ± SD.

**Table 5:** Root Mean Square (RMS, Rq) (nm) roughness values of the 2 × 2 µm^2^ and 5 × 5 µm^2^ topographic images obtained by AFM from each of the films at different percentages of potato starch before and after exposure to UV irradiation.

% (*w*/*v*) Potato Starch	2 × 2 µm^2^		5 × 5 µm^2^	
	Before UV Exposure	After UV Exposure	Before UV Exposure	After UV Exposure
3	13 ± 6 ^a^	13 ± 7 ^a^	29 ± 9 ^a^	31 ± 11 ^a^
5	9 ± 5 ^a^	10 ± 6 ^a^	23 ± 8 ^a^	26 ± 10 ^a^

Similar letters (a) in the row indicate no significant differences between the different films (*p* > 0.05). Data shown correspond to the measurements performed in three areas and represent the mean ± SD.

## Data Availability

The original contributions presented in this study are included in the article. Further inquiries can be directed to the corresponding author.
